# Knowledge, attitude, and practices regarding use of artificial intelligence for medical writings among doctors of Khyber Pakhtunkhwa, Pakistan: a cross-sectional study

**DOI:** 10.1097/MS9.0000000000002953

**Published:** 2025-02-06

**Authors:** Waseem Sajjad, Areeba Inam, Bilal Ahmed, Mohammad Zahir, Ali Mujtaba, Zoha Khan, Muhammad Nabeel Saddique, Javed Iqbal, Jamil Nasrallah

**Affiliations:** a King Edward Medical University, Mayo Hospital, Lahore, Pakistan; b Ayub Medical College, Abbotabad, Pakistan; c Azad Jammu Kashmir Medical College, Muzaffarabad, Pakistan; d Nursing Department, Hamad Medical Corporation, Doha, Qatar; e Faculty of Medical Sciences, Lebanese University, Beirut, Lebanon

**Keywords:** artificial intelligence, doctors, medical writings

## Abstract

**Background and objectives:**

Artificial intelligence (AI) is a cutting-edge technology affecting all eras of science and technology and medicine, which is not an exception. Medical writings play a significant role in improving health standard and patient outcomes owing to their global dissemination. AI has widely affected medical writings. This study aims to determine the knowledge, attitude, and practices among the doctors of Khyber Pakhtunkhwa (KPK) regarding the use of artificial intelligence (AI) in medical writings. Medial writings cover a broad horizon of writings ranging from simple medical reports and prescription and extending to impactful article writings and evidence-based research papers as well as figure and graph generation for these papers using the input data.

**Materials and methods:**

It is a questionnaire-based cross-sectional study involving convenience non-probability sampling. The questionnaire contained a total of 29 items with subsequent portions of knowledge assessment containing 11 items, the attitude assessment comprised 10 items, and the practice assessment involved eight items. Being the first study of its kind, a conservational approach was adopted for sample size determination, and initially, the calculated sample was 385 using an online sample size calculator. The final sample size adopted was 500 to avoid any discrepancies owing to small sample size. The study setting was KPK, a province of Pakistan, and the study population was the medical doctors practicing in KPK. SPSS 28 was used for data analysis. Frequency, percentage, correlation, and regression analysis were used.

**Results:**

Out of 500 respondents, 135 were females and 365 were males. The overall data suggest a high level of knowledge regarding AI in medicine (91.2%). The use of AI was about 30.6%, highlighting a gap that needs to be addressed. The majority of doctors agreed on the impact of AI in medical writing (92.5%), indicating a positive attitude toward promotion within the community. The Pearson coefficient showed statistically significant values. Regression analysis with *P*-value < 0.001 also supported the hypothesis.

**Conclusion:**

The study revealed a positive perception of AI in medical writing, recognizing its potential for efficiency and quality improvement. However, respondents expressed concerns about AI authorship, ethics, privacy, and bias, leading to low adoption. The findings underscore the necessity for education, training, and clear guidelines to responsibly integrate AI into medical writing, ensuring ethical use and advancing the field with informed practices.

**Highlights**

- Doctors in Khyber Pakhtunkhwa, Pakistan, have high knowledge (91.2%) about AI in medicine.
- Despite positive attitudes (92.5%), AI use in medical writing is low (30.6%).
- Concerns about authorship, ethics, privacy, and bias hinder AI adoption.
- Educational programs and clear guidelines are needed for responsible AI integration in medical writing.

## Introduction

Artificial Intelligence is a relatively recent subfield of information technology that performs tasks and activities that normally require the human mind^[1]^. Artificial Intelligence (AI) leverages computers and machines to mimic the problem-solving and decision-making capabilities of a human. AI marks the dawn of a new era that can potentially revolutionize our daily work space. Like all other fields, AI has started invading the medical arena and is now influencing the field both academically and practically, prompting science students to research on the potential of AI in health and writing^[2]^.

AI in medical writings covers a broad horizon of writings ranging from simple medical reports, prescription, presentation, and lab reports and extending to impactful article writings and evidence-based research papers as well as figure and graph generation for these papers using the input data. The use of AI in medical writings and for research purposes is very limited, but according to some reports, AI is playing its part in health care systems, specifically in the field of radiology and to a lesser extent in ophthalmology, psychiatry, cardiology, oncology, neurosciences, pathology, and medicine^[3–8]^. AI has been reported to help researchers with English-language proficiency, literature review, manuscript preparation in the publication process, and bibliography^[9,10]^. Also, there are tools out there like Semantic Scholar and Elicit (Search Engines), TLDR This, Scholarcy, and Genei.io (Summarizing tools), Quilbot’s Citation Generator and Zotero (Citation Generators), and Agent GPT and BabyGPT (Review Literature) that facilitate the cumbersome research process. According to a recent study, 40% of scientists are unaware of the application of Al in a health care setup let alone the medical writing^[2,11]^.

In a developing country like Pakistan, AI is still in its infancy, but some work is worth mentioning. At under- and post-graduate levels, AI subfields of deep and machine learning have been implemented, specially in the medical field. Moreover, it has been tried to integrate AI in social fabric on the state level under a president initiative, but it faced problems in health care set ups, mainly due to low resources, inadequate professional training of algorithms, physicians’ fear of AI’s domination, and social barriers^[5,12,13]^.

It has been found that very limited research work is done on the attitude of physicians and use of AI in medical writings. Though the study has been done in some developing countries like Syria and even a recent study done in Pakistan on this topic depicts 74% knowledge of AI, 27.3% awareness regarding AI use in medicine, and 76.7% positive attitude among doctors of Pakistan, a study like this has not been carried out in the study setting of Khyber Pakhtunkhwa province of Pakistan, which provides a research gap to this study, and the results can dictate the intensity of steps that need to be taken in order to inculcate the awareness and use of AI by physicians of KP in medicine and writings to keep up with the first-world technologies^[14,15]^.

Keeping the significance of this study and the vivid research gap as mentioned above, this study is aimed to comprehensively evaluate various aspects related to the incorporation of artificial intelligence (AI) in medical writings among doctors in Khyber Pakhtunkhwa. First, the investigation sought to assess the level of knowledge that doctors in the region possess concerning the application of AI in medical writings. Subsequently, the study aimed to delve into the attitudes held by doctors in Khyber Pakhtunkhwa toward the utilization of AI in their medical writings. Furthermore, the research sought to investigate the current practices of doctors in the region in terms of integrating AI into their medical writings. Another key objective was to identify both the perceived benefits and challenges associated with the use of AI in medical writings among doctors in Khyber Pakhtunkhwa. Finally, the study aimed to discern the various factors that influence the adoption of AI in medical writings by doctors in the specified region. This comprehensive approach was designed to provide a nuanced understanding of the multifaceted dimensions surrounding the integration of AI in medical practices among healthcare professionals in Khyber Pakhtunkhwa.

## Materials and methods

The research employed a cross-sectional study design conducted in Khyber Pakhtunkhwa, Pakistan, focusing on the doctor population in the region. The study utilized a convenience, non-probability sampling technique to select participants. We have adopted this sampling technique owing to our limited resources and access. Though this sampling technique may cause the potential limitation of generalizability due to lack of randomization factors, a gross overview of this innovative addition to medical writings can definitely be well-demonstrated by the findings of this study.

Inclusion criteria comprised doctors practicing in Khyber Pakhtunkhwa, covering various specialties and professional settings, proficient in English, and possessing diverse levels of experience, including both junior and senior doctors. Additionally, participants were required to have at least one published paper. Exclusion criteria excluded non-doctors, individuals not actively practicing medicine in Khyber Pakhtunkhwa, those facing language barriers, incomplete survey responders, and doctors without any published papers. We specifically considered the population of Khyber Pakhtunkhwa because majority of the collaborators of this project belong to Khyber Pakhtunkhwa which made the process of data collection form this very specific population of doctors easy and practical. Moreover, this region also reflects a diverse representation of healthcare professionals working in both urban and rural settings, making the findings more relevant to similar healthcare systems and enhance the generalizability of results keeping the economic burden of study reasonable. However, this can also be a limitation factor of the study as we have mentioned in the discussion section of the study.

Being the first of its kind, the study used a conservative approach to the sample size, and initially, the sample size was determined to be 385, but later on, a sample size was increased to 500 to enhance the quality of the study and decrease any accidental discrepancies owing to small sampling techniques and lack of randomization in the adapted sampling technique. In general, a large sample size can improve research results by increasing precision estimates, enhancing statistical power, and providing more reliable and valid results^[16]^.

Ethical approval was obtained from the Ethical Review Board (ERB) after presenting the synopsis of the project to ERB. Data collection involved the distribution of a questionnaire, developed and tested by investigators. The questionnaire comprised five distinct sections. The initial segment focused on collecting demographic data from participants, encompassing variables such as age, gender, institution classification (public or private), and designation categorization. The subsequent sections were dedicated to evaluating participants’ knowledge, attitudes, and practices (KAP) regarding artificial intelligence (AI) among doctors in the Khyber Pakhtunkhwa region of Pakistan. The knowledge assessment portion encompassed 11 items, the attitude assessment comprised 10 items, and the practice assessment involved eight items. A panel of experts, comprising four professors specializing in medicine, validated the questionnaire’s content. To ensure the clarity of all questions in the instrument, a pilot study involving 30 doctors was undertaken, and their data were excluded from the final analysis. The internal consistency of the three latent variables –knowledge, attitude, and practice – was verified through the calculation of Cronbach’s alpha. The feedback response from the following study urged us to make minor changes in the questionnaire to improve the clarity of the statement and streamline the flow; however, no structural changes or significant modification was made to mention. Some statements’ ambiguities were cleared through changing words to make them easily understandable so that the participants can answer them without any confusion or misunderstanding, preventing the results from any bias caused by unclear statements and complex statements as the questionnaire was to be self-filled by the participants without any interpreter among the investigator or any other third person on the behalf of investigators. The questionnaire, accompanied by implied consent, was administered to eligible participants. Participants were offered the questionnaire in both hard copy and online through Google Forms.

The analysis of the data was conducted utilizing SPSS version 28.0. Categorical variables were presented using frequencies and percentages, while continuous variables were represented by the median with a corresponding 95% confidence interval. To elucidate the factors associated with knowledge, attitudes, and practices (KAP) scores, three quantile regression models were developed. The knowledge-score model incorporated independent variables such as age, gender, institute type (private vs. public), and designation. The attitude score model included the aforementioned variables along with the knowledge score as additional independent variables. Furthermore, the practice-score model encompassed both knowledge and attitude scores as independent variables. The significance level for all analyses was set at *P* < 0.05. This study is conducted in strict compliance with the STORCSS guidelines.

## Results

### Demographic stats

The data present insights into the demographic and professional characteristics of the participants in our survey. Among the 500 participants, 73.0% were male, while 27.0% were female. Regarding the institutions where participants worked, a significant majority, comprising 85.8%, were affiliated with public hospitals or institutes, while the remaining 14.2% were associated with private institutions. In terms of departmental distribution within the medical field, 6.8% of participants were involved in basic science, while the majority, 56.8%, were engaged in medicine and allied fields, and 36.5% in surgery and allied fields. Concerning working status or designation, the participants held various roles, with 45.9% being house officers, 12.8% medical officers, 19.6% demonstrators, and 21.6% post-graduate residents. These findings are shown in Table 1 for further understanding and summarization.

**Table 1:** Demographic stats

Variable	Frequency	Percent
Gender of the participant		
Male	365	73.0
Female	135	27.0
Institute/Hospital here the participant works		
Public	432	85.8
Private	68	14.2
Department in medical field		
Basic science	34	6.8
Medicine and allied	284	56.8
Surgery and allied	196	36.5
Working status or designation of the participant		
House officer	230	45.9
Medical officer	64	12.8
Demonstrator	57	19.6
Post-graduate resident	108	21.6

### Knowledge stats

**Figure 1.**
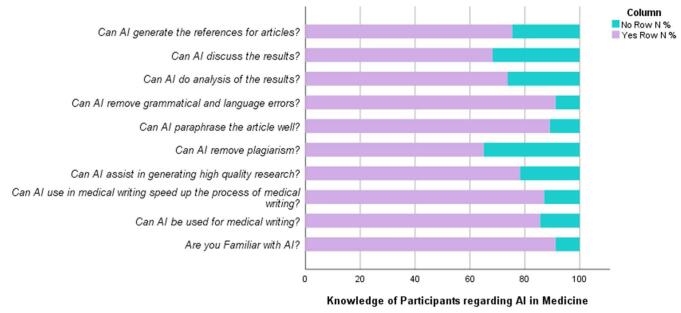
Knowledge of participants regarding AI in medicine.

Table 2 provides insights into the familiarity and perceptions regarding the use of artificial intelligence (AI) for medical writing among respondents. It shows that a vast majority, 91.2%, of participants are familiar with AI, indicating a high level of awareness within the surveyed group. Moreover, the data reveal positive attitudes toward AI’s potential in medical writing, with substantial percentages believing in its utility. Specifically, 85.5% acknowledge the possibility of using AI for medical writing, while 87.0% believe it can expedite the writing process. Additionally, a significant proportion, 78.2%, see AI as capable of aiding in the generation of high-quality research. Regarding specific functions, a majority of respondents believe AI can perform various tasks effectively. For instance, 65.0% agree that AI can remove plagiarism, and 89.0% believe it can paraphrase articles proficiently. Moreover, 91.2% believe AI can eliminate grammatical and language errors, indicating confidence in its editing capabilities. However, there are slightly lower percentages for tasks involving analysis and discussion of results, with 73.8% believing AI can analyze results and 68.3% believing it can discuss them. Nonetheless, a significant proportion, 75.3%, agree that AI can generate references for articles. The data also provide insights into the sources from which respondents acquired information about AI for medical writing. Social media emerges as the most prominent source, cited by 48.7% of respondents, followed by friends (17.6%) and colleagues (14.2%). Fig. 1. shows the above-mentioned stats. Conferences and seminars, along with other unspecified sources, also contribute to respondents’ knowledge about AI in medical writing.

**Table 2:** Knowledge stats

Variable	Yes (%)	No (%)
Are you familiar with AI?	91.2%	8.8%
Can AI be used for medical writing?	85.5%	14.5%
Can AI use in medical writing speed up the process of medical writing?	87.0%	13.0%
Can AI assist in generating high-quality research?	78.2%	21.8%
Can AI remove plagiarism?	65.0%	35.0%
Can AI paraphrase the article well?	89.0%	11.0%
Can AI remove grammatical and language errors?	91.2%	8.8%
Can AI analyze the results?	73.8%	26.2%
Can AI discuss the results?	68.3%	31.7%
Can AI generate the references for articles?	75.3%	24.7%
From which source did you get information about use of AI for medical writing?		
Friends	17.6	
Colleague	14.2	
Social media	48.7	
Conferences and Seminars	4.7	
Other	14.9	

### Attitude stats

**Figure 2.**
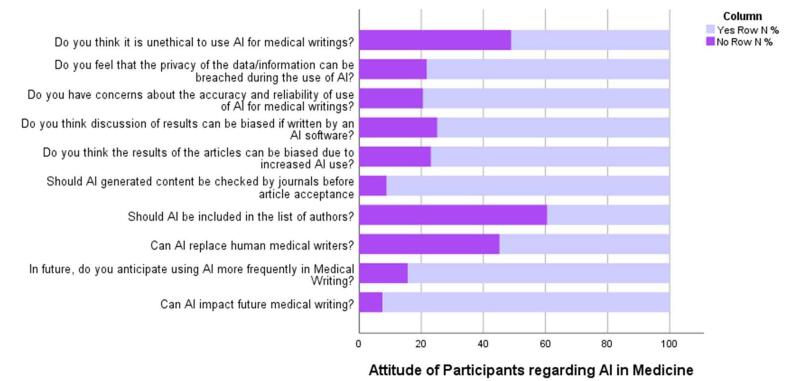
Attitude of participants regarding AI in medicine.

The survey results offer a comprehensive insight into the perceptions and attitudes of respondents toward the integration of artificial intelligence (AI) in the realm of medical writing. A prevailing sentiment emerges, with a resounding 92.5% expressing belief in the potential impact of AI on the future of medical writing. This optimism extends to the practical realm, as 84.4% anticipate an increased utilization of AI in their medical writing practices. However, a nuanced perspective emerges regarding the role of AI alongside human authors, with 54.8% acknowledging the possibility of AI replacing human medical writers, while 45.2% remain skeptical. Ethical considerations come to the forefront, as reflected in the responses. While 39.5% support the inclusion of AI as authors in medical publications, a majority of 60.5% express reservations about such a proposition. Concerns about quality control are evident, as 91.2% advocate for journals to scrutinize AI-generated content before acceptance, emphasizing the importance of maintaining rigorous editorial standards. The results also highlight apprehensions regarding potential biases introduced by AI, both in the results (76.9%) and the discussion thereof (74.8%). These concerns underscore the need for vigilance in ensuring unbiased and accurate representation in AI-assisted medical writing. Moreover, the survey captures a dual perspective on the ethical implications of AI in medical writing, as exactly 51.0% consider its use unethical, revealing a split in ethical stances. Privacy concerns also emerge, with 78.2% expressing worries about potential data breaches during the use of AI. Last, respondents express varying degrees of acceptance regarding the percentage of AI content in medical writing, with 54.7% indicating approval for less than 20% and 45.3% open to a higher proportion. Fig. 2 displays the findings discussed above. Table 3 shows the respective findings.

**Table 3:** Attitude stats

Variable	Yes (%)	No (%)
Can AI impact future medical writing?	92.5%	7.5%
In future, do you anticipate using AI more frequently in medical writing?	84.4%	15.6%
Can AI replace human medical writers?	54.8%	45.2%
Should AI be included in the list of authors?	39.5%	60.5%
Should journals check AI-generated content before article acceptance?	91.2%	8.8%
Do you think the results of the articles can be biased due to increased AI use?	76.9%	23.1%
Do you think a discussion of results can be biased if written by AI software?	74.8%	25.2%
Do you have concerns about the accuracy and reliability of the use of AI for medical writing?	79.5%	20.5%
Do you feel that the privacy of the data/information can be breached during the use of AI?	78.2%	21.8%
Do you think it is unethical to use AI for medical writings?	51.0%	49.0%
What percentage of AI content shall be allowed?		
Less than 20%	54.7	
Greater than 20%	45.3	

### Practice stats

**Figure 3.**
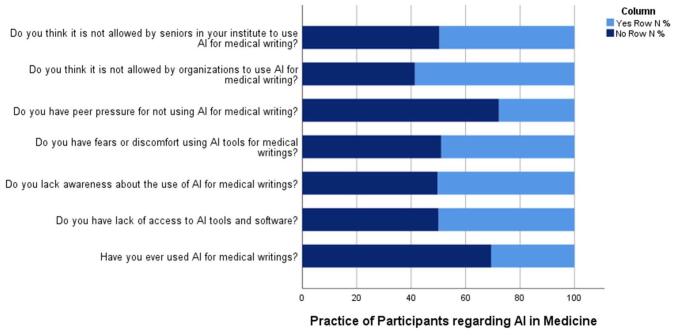
Practices of participants regarding AI in medicine.

The survey results shed light on the usage patterns, attitudes, and perceptions regarding the utilization of artificial intelligence (AI) in medical writing among respondents. A minority, comprising 30.6% of participants, reported having used AI for medical writings, indicating a relatively low adoption rate within the surveyed group. Among those who have used AI, paraphrasing emerged as the primary purpose for 47.3%, followed by result discussion and referring, indicating a diverse range of applications for AI tools in medical writing. Interestingly, a significant proportion, 50.3%, admitted to lacking awareness about the use of AI for medical writings, highlighting the need for increased education and training on AI applications in this domain. Moreover, nearly half of the respondents (49.0%) expressed fears or discomfort regarding the use of AI tools for medical writing, indicating apprehension or uncertainty about its efficacy or implications. Peer pressure appears to play a role in shaping attitudes toward AI adoption, as 27.8% reported feeling pressure not to use AI for medical writing, suggesting potential social dynamics influencing individual decisions. Moreover, a substantial percentage, 58.6%, believes that organizations do not permit the use of AI for medical writing, indicating perceived barriers or restrictions within institutional contexts. Interestingly, respondents also expressed uncertainty about the acceptance of AI among senior professionals in their institutes, with an almost equal split between those who believe it is allowed (49.7%) and those who do not (50.3%). This finding reflects a complex interplay of organizational policies, cultural norms, and individual perceptions regarding the use of AI in medical writing. Findings regarding practice of AI are depicted in Fig. 3. Table 4 shows the above-mentioned data.

**Table 4:** Practice stats

Variable	Yes (%)	No (%)
Have you ever used AI for medical writings?	30.6%	69.4%
What was your main purpose for using AI in medical writing:		
Results discussion	12.8	
Referring	12.2	
Paraphrasing	47.3	
Other	27.7	
Do you lack awareness about the use of AI for medical writings?	50.3%	49.7%
Do you have fears or discomfort using AI tools for medical writings?	49.0%	51.0%
Do you have peer pressure for not using AI for medical writing?	27.8%	72.2%
Do you think it is not allowed by organizations to use AI for medical writing?	58.6%	41.4%
Do you think it is not allowed for seniors in your institute to use AI for medical writing?	49.7%	50.3%

### Correlation analysis

**Figure 4.**
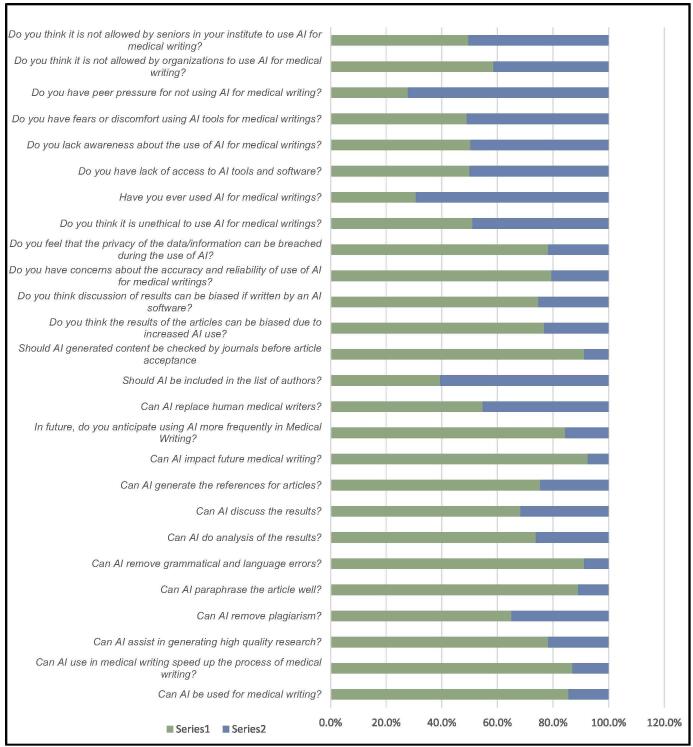
Stacked bar chart of knowledge, attitude, and practice analysis.

The correlation statistics provided offer insights into the relationships between knowledge, attitude, and practice regarding the use of artificial intelligence (AI) in medical writing. The data reveal several notable patterns. First, there is a positive correlation between knowledge and attitude, as indicated by a Pearson correlation coefficient of 0.410, which is statistically significant at the 0.01 level. This suggests that individuals with greater knowledge about AI in medical writing tend to have more positive attitudes toward its usage. Similarly, a positive correlation is observed between attitude and practice, with a Pearson correlation coefficient of 0.315, also significant at the 0.01 level. This indicates that individuals with favorable attitudes toward AI in medical writing are more likely to engage in practices that involve its utilization. Additionally, a positive correlation is evident between knowledge and practice, although to a slightly lesser extent compared to knowledge-attitude and attitude-practice correlations. The Pearson correlation coefficient between knowledge and practice stands at 0.275, significant at the 0.01 level. This suggests that individuals with higher levels of knowledge about AI in medical writing are more inclined to incorporate it into their practices. Overall, the correlation statistics underscore the interconnectedness between knowledge, attitude, and practice regarding the use of AI in medical writing. They suggest that increasing knowledge and fostering positive attitudes toward AI can potentially lead to greater integration of AI tools into medical writing practices. These findings highlight the importance of education, training, and fostering favorable attitudes in promoting the effective adoption of AI in the medical writing domain (Fig. 4). The stats are shown in Table 5.

**Table 5:** Correlation analysis

*Correlations*				
		Knowledge	Attitude	Practice
Knowledge	Pearson correlation	1		
	Sig. (2-tailed)			
Attitude	Pearson correlation	0.410^a^	1	
	Sig. (2-tailed)	0.000		
Practice	Pearson correlation	0.275^a^	0.315^a^	1
	Sig. (2-tailed)	0.001	0.000	

^a^. Correlation is significant at the 0.01 level (2-tailed).

The correlation of familiarity with artificial intelligence (AI) and its use in medical writings could also be seen. The Pearson correlation coefficient between these two variables is 0.088, indicating a very weak positive relationship. This suggests that individuals who are familiar with AI are only slightly more likely to have used it for medical writing, though this relationship is almost negligible.

The significance (*P*-value) of 0.292 is greater than the standard threshold of 0.05, meaning the relationship is not statistically significant. In other words, the weak correlation could be due to random chance, and familiarity with AI does not significantly influence whether someone has used it for medical writing in this sample. The number of participants analyzed was 147 for familiarity with AI and 146 for AI usage in medical writing. Overall, there is no strong or significant relationship between these two factors in the dataset. The above-mentioned stats are shown in Table 6.

**Table 6:** Correlation analysis

Correlations			
		Familiarity with AI	Have you ever used AI for medical writing?
Familiarity with AI	Pearson correlation	1	
Have you ever used AI for medical writing?	Pearson correlation	.088	1

### Regression analysis

The provided regression results in Table 7 reveal valuable insights into the relationships between attitude, knowledge, and practice regarding the integration of artificial intelligence (AI) in medical writing. The regression analysis focuses on the impact of attitude, treated as the independent variable, on both knowledge and practice, serving as dependent variables. For the variable “knowledge,” the beta coefficient of 0.293 signifies a positive relationship between attitude and knowledge. This coefficient, combined with the R2 value of 0.179, suggests that attitude explains approximately 17.9% of the variance in knowledge. The F-statistic of 15.650 is statistically significant at *P* < 0.001, indicating that the relationship between attitude and knowledge does not occur by random chance. This finding implies that a more positive attitude toward AI in medical writing is associated with higher levels of knowledge about the subject. Similarly, for the variable “practice,” the beta coefficient of 0.235 indicates a positive association between attitude and practice. The R2 value of 0.179 suggests that attitude explains around 17.9% of the variance in practice. The F-statistic of 15.650 is statistically significant at *P* = 0.003, reinforcing that the observed relationship between attitude and practice is unlikely to be coincidental. Thus, a favorable attitude toward AI in medical writing appears to be linked with a higher likelihood of incorporating AI tools into actual writing practices. In summary, these regression results suggest that attitude significantly influences both knowledge and practice in the context of AI adoption in medical writing. Professionals with more positive attitudes are likely to possess greater knowledge about AI and are more inclined to integrate AI into their actual writing practices. These findings emphasize the importance of cultivating positive attitudes as a potential driver for knowledge acquisition and practical implementation of AI tools in the field of medical writing.

**Table 7:** Regression analysis of predictors^*^ against dependent variable^**^

Variable	Beta coefficient	R^2^	F	t-value	*P*-value
**Knowledge**	0.293	0.179	15.650	**3.730**	0.000
**Practice**	0.235	0.179	15.650	**2.985**	0.003

^*^ Predictors: (constant) knowledge, practice.

^**^ Dependent variable: attitude.

## Discussion

In this cross-sectional study, we found a positive attitude and perceptions of medical students and healthcare professionals in the use of artificial intelligence (AI) in medical and scientific writings. The majority of the participants acknowledged the potential and capabilities of AI and expressed a welcoming attitude toward its use in paraphrasing, manuscript writing, translation, data analysis, editing, and publication processes.

On the other hand, a number of participants also expressed reservations and concerns regarding its ethical implications, data privacy, potential of bias in AI-generated content, and matter of AI authorship. We also found that there is low-adoption and use of AI tools in medical writing due to lack of awareness, potential restrictions, and low credibility and acceptance of AI-generated content. It is because the AI tools are currently in developmental stages and need some time for their critical appraisal and validity of AI-generated content.

We also found that the majority of the respondents agreed that there is a dire need of development of guidelines and regulations which ensure ethical and responsible use of AI in medical writing. We found that there is judicious need to develop and monitor user-friendly and ethically compliant tools to ensure and uphold high-quality scientific literature. To address these ethical concerns regarding the use of AI in medical writings, the use of AI in medical writings should be evaluated by experts and clear rules and regulations should be introduced. The questions regarding the bias, privacy, and authorship should be addressed properly. Bias remains a significant risk, as AI models may inadvertently perpetuate existing disparities in healthcare literature due to the data they are trained on. This raises questions about the fairness and reliability of AI-generated content, especially in fields where impartiality is critical. Additionally, privacy concerns arise from the potential misuse of sensitive medical information, underscoring the need for stringent data security measures to safeguard patient confidentiality. Last, the apprehension about authorship should be debated and settled through clearcut policy and rules, as reliance on AI challenges traditional notions of intellectual ownership and accountability in academic writing. Addressing these ethical dimensions is crucial to ensure responsible integration of AI in medical literature while maintaining trust in scientific integrity.

Similar findings were reported in a scoping review which states that there are interests as well as worries concerning use of AI in generating medical literature. This study found that AI tools can help in doing a robust literature review, managing references, performing data analysis, decreasing time to write more effective and faster manuscripts, analyzing and summarizing data from multiple sources, detecting plagiarism, etc. It also reported concerns about the use of AI regarding inaccurate and malicious non-existent information, fabrication, and potential of bias in judgment^[17]^.

A study conducted at a university revealed most of the students (41%) had moderate to good knowledge and attitude toward AI and its application in medical education and practice. Over 80% of students emphasized the need to integrate teaching about AI in their medical curricula^[18]^. While another study reported that 56.7% of the participants had a good familiarity with artificial intelligence and 57.9% of the participants agreed that the biggest advantage of using artificial intelligence in healthcare was its ability to speed up processes^[19]^.

Another study determined knowledge, attitude, and practice of AI among doctors and medical students in Syria. It found that most participants have positive attitudes toward AI in the medical field. It revealed that 70% of participants had previous knowledge about AI and only 23.7% of participants knew about AI’s application in the medical field^[14]^. A similar study conducted in Nepal found that the undergraduate medical students have a low understanding of AI and its implications for healthcare. It also stated that the medical curricula in Nepal lack AI-related education. The respondents had a poor perception of AI. The median knowledge score toward AI was 11^[20]^.

An online survey-based cross-sectional stated that there is lack of knowledge about AI and its applications among doctors and medical students in Pakistan. It found 74% of doctors and 68.8% of medical students had basic knowledge of AI. Only 27.3% of doctors and 19.4% of students were aware of its medical applications. However, they have a positive attitude toward AI in medicine and are willing to adopt it^[15]^. It was also reported in a study that gender disparities and lack of knowledge are also found in medical students and doctors regarding AI in healthcare. It emphasized that formal training courses are needed to teach AI in medical schools and hospitals^[21]^.

Moreover, two online surveys were conducted in Germany in 2021. A total of 1,001 and 1,000 adults, respectively, participated in the surveys. It also found that healthcare professionals are reluctant to implement AI-powered devices and tools. The survey results highlight the need to improve education and perception of medical AI applications including medical writing.

While a majority of respondents acknowledge the potential of AI and express positive attitudes toward its use, there are also concerns about ethical considerations, data privacy, and potential biases. The findings highlight the need for increased education and training on AI applications in medical writing, as well as the importance of addressing ethical concerns and ensuring data privacy. As AI technology continues to evolve, it is likely to play an increasingly important role in medical writing. However, it is crucial to ensure that AI is used responsibly and ethically and that human oversight remains essential.

Though our study showed a higher level of awareness of AI in medical writings, they in contrast showed a low level of adoptability to these tools. This may be attributed to their access to these tools as most of the tools of AI are paid and the one that is free is not that reliable and precise. Moreover, institutional policies and individual perception regarding the ethical considerations are also considered in this regard. No doubt, a steep adaptation spike has been seen in the use of AI in medical writings; however, many questions regarding this use are still unanswered globally like the authorship attributions to AI, etc. This may also be a factor to the low adaptation despite high awareness. However, further research can be warned in this regard.

## Limitations

This study has several limitations that should be considered when interpreting the findings. First, the sample size was relatively small and may not be generalizable to the wider population of medical professionals. Second, the study relied on self-reported data, which can be subject to bias. Third, the study did not explore the long-term impact of AI on medical writing practices. Fourth, the sampling technique was of non-probability, and the probability-type sampling may result in somewhat different results. Further research is needed to address these limitations and provide a more comprehensive understanding of the use of AI in medical writing. While this method is pragmatic for initial studies in this field, it may introduce selection bias and limit the generalizability of the findings owing to its non-randomized and non-stratified recruitment of participants into study. This may give results affected by regional and institutional policies and access of the participants to the resources, i.e., tools of AI in medical writings.

## Future prospects and recommendations

Despite the limitations, this study suggests that AI has the potential to play a significant role in the future of medical writing. As AI technology continues to develop, it is likely that AI tools will become more sophisticated and be integrated into medical writing practices. There is need to develop and evaluate user-friendly, ethically sound tools that address the specific writing needs. However, it is important to address the ethical concerns and ensure that AI is used responsibly and transparently. The development of clear guidelines and regulations, as well as ongoing education and training for medical professionals, will be essential to ensure the successful and ethical adoption of AI in medical writing.

As the world is progressing toward practicality of artificial intelligence in every area of human lives, it is imperative that the rising concerns should be adequately addressed on logical and understanding basis as well as augmented by specified rules and regulations. This will not only reduce the burden of concerns regarding its use in medical writings but will also enhance the adaptability of AI in medical writings without the fear of any concerns.

Also, it is important to integrate and collaborate with the AI tool development information and artificial technology companies and developers to address the potential concerns. They should clearly communicate to users where the data they acquire from users are used and how privacy is maintained. Also, a most important factor that is not yet properly addressed by major AI tools providing companies is the lack of mentioning the clear source of the information they provide the users and the guidelines behind that. If AI-providing companies address all these concerns, it can lead to better adoptability of AI in medical writing.

## Conclusion

The study revealed a positive perception of AI in medical writing, recognizing its potential for efficiency and quality improvement with a majority of respondents acknowledging its potential in expediting writing, improving quality and efficiency, and aiding in complex tasks like paraphrasing. However, respondents expressed concerns about AI authorship, ethics, privacy, and bias, leading to low adoption. The findings underscore the necessity for education, training, and clear guidelines to responsibly integrate AI into medical writing, ensuring ethical use and advancing the field with informed practices. We also found a low adoption of AI in medical writing which may be attributed to the above-mentioned concerns and potential organizational restrictions. This study highlights the need for further education and training on AI applications in medical writing, as well as the development of clear guidelines and regulations to ensure responsible and ethical use of AI in this domain.

Based on our findings, we will suggest actionable steps for policymakers, such as promoting awareness campaigns and creating guidelines for responsible AI integration in medical writings. For educational institutions, we will recommend incorporating AI-related training into medical curricula to enhance the effective use of AI in medical writing and practice. This will not only increase the awareness but will help future doctors to adapt these tools in their practices.

## Data Availability

The participants of this study did not give written consent for their data to be shared publicly, so due to the sensitive nature of the research, supporting data are not available.

## References

[1] Artificial Intelligence (AI): What Is AI and How Does It Work? | Built In [Internet]. [cited 2023 July 12]. Available from: https://builtin.com/artificial-intelligence

[2] Parisis N. Medical writing in the era of artificial intelligence. Medical Writing 2019;28:4–9.

[3] Waymel Q Badr S Demondion X. Impact of the rise of artificial intelligence in radiology: what do radiologists think? Diagn Interv Imaging. 2019;100:327–36. 31072803 10.1016/j.diii.2019.03.015

[4] Sarwar: physician perspectives on integration of. Google Scholar [Internet]. [cited 2023 July 12]. Available from: https://scholar.google.com/scholar_lookup?title=Physician%20perspectives%20on%20integration%20of%20artificial%20intelligence%20into%20diagnostic%20pathology&author=S.%20Sarwar&publication_year=2019&pages=1-7

[5] Guo J Li B. The application of medical artificial intelligence technology in rural areas of developing countries. Health Equity. 2018;2:174–81. 30283865 10.1089/heq.2018.0037 PMC6110188

[6] Sit C Srinivasan R Amlani A. Attitudes and perceptions of UK medical students towards artificial intelligence and radiology: a multicentre survey. Insights Imaging 2020;11:14. 32025951 10.1186/s13244-019-0830-7 PMC7002761

[7] Shimizu H Nakayama KI. Artificial intelligence in oncology. Cancer Sci 2020;111:1452–60. 32133724 10.1111/cas.14377 PMC7226189

[8] Hassabis D Kumaran D Summerfield C. Neuroscience-inspired artificial intelligence. Neuron. 2017;95:245–58. 28728020 10.1016/j.neuron.2017.06.011

[9] Using AI to improve the medical writing experience [Internet]. [cited 2023 July 12]. Available from: https://thejournalofmhealth.com/using-ai-to-improve-the-medical-writing-experience/

[10] Biswas S. ChatGPT and the future of medical writing. Radiology. 2023;307:e223312. 36728748 10.1148/radiol.223312

[11] “Essential AI Tools every medical writer” | search | linkedIn [Internet]. [cited 2023 July 12]. Available from: https://www.linkedin.com/search/results/all/?keywords=essentail%20ai%20tools%20every%20medical%20writer&origin=GLOBAL_SEARCH_HEADER&sid=2i%3A

[12] Abid S Awan B Ismail T. Artificial intelligence: medical student s attitude in district Peshawar Pakistan. PJPH 2019;9:19–21.

[13] Chan KS Zary N. Applications and challenges of implementing artificial intelligence in medical education: integrative review. JMIR Med Educ. 2019;5:e13930. 31199295 10.2196/13930 PMC6598417

[14] Swed S Alibrahim H Elkalagi NKH. Knowledge, attitude, and practice of artificial intelligence among doctors and medical students in Syria: a cross-sectional online survey. Front Artif Intell 2022;5:1011524. 36248622 10.3389/frai.2022.1011524 PMC9558737

[15] Ahmed Z Bhinder KK Tariq A. Knowledge, attitude, and practice of artificial intelligence among doctors and medical students in Pakistan: a cross-sectional online survey. Ann Med Surg (Lond) 2022;76:103493. 35308436 10.1016/j.amsu.2022.103493 PMC8928127

[16] Sink CA Mvududu NH. Statistical power, sampling, and effect sizes: three keys to research relevancy. Couns Outcome Res Eval 2010;1:1–18.

[17] Khalifa AA Ibrahim MA Artificial intelligence (AI) and ChatGPT involvement in scientific and medical writing, a new concern for researchers. A scoping review. AGJSR [Internet]. 2024 January 4; [cited 2024 Feb 25]. Available from: https://www.emerald.com/insight/content/doi/10.1108/AGJSR-09-2023-0423/full/html

[18] Khater AS Zaaqoq AA Wahdan MM. Knowledge and attitude of ain shams university medical students towards artificial intelligence and its application in medical education and practice. Educ Res Innov J. 2023;3:29–42.

[19] Ahmer H Bin Altaf S Mustufa Khan H. Knowledge and perception of medical students towards the use of artificial intelligence in healthcare. J Pak Med Assoc 2023;73:448–51. 36800756 10.47391/JPMA.5717

[20] Jha N Shankar PR Al-Betar MA. Undergraduate medical students’ and interns’ knowledge and perception of artificial intelligence in medicine. AMEP 2022;13:927–37. 10.2147/AMEP.S368519 PMC9419901 36039185

[21] Kansal R Bawa A Bansal A. Differences in knowledge and perspectives on the usage of artificial intelligence among doctors and medical students of a developing country: a cross-sectional study. Cureus [Internet]. 2022 Jan 19; [cited 2024 February 26]; Available from: https://www.cureus.com/articles/83661-differences-in-knowledge-and-perspectives-on-the-usage-of-artificial-intelligence-among-doctors-and-medical-students-of-a-developing-country-a-cross-sectional-study 10.7759/cureus.21434 PMC8860704 35223222

